# Uncertainty quantification of dynamic earthquake rupture simulations

**DOI:** 10.1098/rsta.2020.0076

**Published:** 2021-05-17

**Authors:** Eric G. Daub, Hamid Arabnejad, Imran Mahmood, Derek Groen

**Affiliations:** ^1^ Research Engineering Group, Alan Turing Institute, London, UK; ^2^ Department of Computer Science, Brunel University London, London, UK

**Keywords:** uncertainty quantification, earthquake mechanics, model calibration, simulation management

## Abstract

We present a tutorial demonstration using a surrogate-model based uncertainty quantification (UQ) approach to study dynamic earthquake rupture on a rough fault surface. The UQ approach performs model calibration where we choose simulation points, fit and validate an approximate surrogate model or emulator, and then examine the input space to see which inputs can be ruled out from the data. Our approach relies on the mogp_emulator package to perform model calibration, and the FabSim3 component from the VECMA toolkit to streamline the workflow, enabling users to manage the workflow using the command line to curate reproducible simulations on local and remote resources. The tools in this tutorial provide an example template that allows domain researchers that are not necessarily experts in the underlying methods to apply them to complex problems. We illustrate the use of the package by applying the methods to dynamic earthquake rupture, which solves the elastic wave equation for the size of an earthquake and the resulting ground shaking based on the stress tensor in the Earth. We show through the tutorial results that the method is able to rule out large portions of the input parameter space, which could lead to new ways to constrain the stress tensor in the Earth based on earthquake observations.

This article is part of the theme issue ‘Reliability and reproducibility in computational science: implementing verification, validation and uncertainty quantification *in silico*’.

## 1. Introduction

Scientists frequently use computer simulations to study complex phenomena that are poorly constrained by observational data such as climate [1], earthquakes [2], tsunamis [3] and other physical systems. These computer simulations usually involve solving complex partial differential equations, and due to the computational cost such simulations can rarely be run at the resolution needed to capture all of the relevant physics.

Because of this, simulations have to capture missing physics in an often ad hoc way, and it is difficult to calibrate and estimate parameters for these models directly [4]. This poses a challenge, as there is a high-dimensional input space from which only a small subset of parameter choices can plausibly reproduce the observational data, while only a limited number of model evaluations are computationally feasible.

A common approach to interrogate the real world using these models is to run a limited ensemble of simulations and fit a *surrogate* model (also referred to in some contexts as an *emulator*) that is able to approximate the expensive simulation [5]. This is frequently done using Gaussian process (GP) regression to approximate the simulations [6], as GPs can be flexibly specified, are straightforward to fit using standard linear algebra procedures and provide robust error estimates of their predictions. The GP emulator is then used to query densely from the input space to carry out model calibration and choose plausible inputs for the simulation.

This work explores use of a software library designed to carry out surrogate model calibration, mogp_emulator (Multi-Output Gaussian Process Emulator, the core surrogate model in this workflow), which implements the procedures described in this work in addition to a number of other techniques. While we focus on this approach in this paper, other uncertainty quantification (UQ) approaches can be used to examine the outputs of the simulations shown here. For instance, the VECMA toolkit [7] also has a UQ library EasyVVUQ [8], which can draw samples and collate experimental runs in addition to carrying out a number of UQ approaches, which are complementary to the focus in this study on model calibration. For instance, another approach to UQ would be to conduct a sensitivity analysis of the simulation outputs [9], which aims at determining how the output variability is related to the various simulation inputs. This information is complementary to the calibration results, and could provide additional information on how to further explore the parameter space with additional simulation runs.

However, while surrogate modelling approaches are common among statistics researchers, they are less frequently used by the domain experts that develop and run the physical models. Because of this, simulation studies often do not conduct rigorous UQ on their outputs, and parameter selection is often performed by hand-tuning or trial-and-error approaches due to the high computational expense of the underlying simulation. A major goal of the software libraries used in this paper is to facilitate domain experts performing UQ on simulation outputs without needing an in-depth understanding of the underlying statistical methods.

This problem additionally presents a significant computational challenge, as it requires generating samples and collating the results of a potentially large number of high-performance computer simulations. Researchers may need to carry out simulations on a number of different computational resources at different resolutions, which poses a problem for reproducibility. To manage this problem, we use FabSim3 [10] to generate templates for the various pieces of this work, from drawing samples and carrying out the simulations to analysing the results.

In this paper, we implement a comprehensive UQ calibration workflow and manage an ensemble of simulations of a dynamic earthquake rupture. Dynamic earthquake rupture is a challenging, multi-scale simulation problem, and due to the fact that earthquakes typically occur at around 10 km depth, seismologists can usually only rely on seismic waves at the surface to constrain the rupture process. Because of this, we do not completely understand the relevant physics for modelling frictional failure [11]. However, while simulations have increasingly been used to understand ground motions and seismic hazard [12,13], a full calibration approach like the one described in this paper has not to our knowledge been previously conducted.

In the following sections, we describe the UQ approach, provide details on the earthquake simulation model, and finally discuss our approach for automating the workflow. We then show the results of the experimental design, surrogate modelling and calibration of an earthquake model. The work presented here was originally conceived as a tutorial for participants at the ‘Reliability and reproducibility in computational science: Implementing verification, validation and uncertainty quantification in silico’ workshop held at the Alan Turing Institute on 24 January 2020. The FabSim3 plugin was used in a tutorial and we provided a pre-packaged computational environment to allow users to re-create the workflows here during a 90 min session. We found that most users were able to complete the exercises within the session and reproduce our results, illustrating the effectiveness of our approach for capturing a full complex UQ workflow in a reliable and reproducible manner. Based on this experience, we view this work as a tutorial demonstration that highlights some of the computational issues involved with UQ, and have simplified the simulation complexity and cost, as well as some details of the calibration workflow, in order to make this more accessible to a wider audience. We have also noted several places where we have made some simplifications and a more careful consideration would be warranted when applying this workflow in a research setting. We feel this work illustrates ways that existing software libraries can help address these problems, while simultaneously highlighting some of the challenges by applying it to a real problem in earthquake science.

## 2. Uncertainty quantification approach

In UQ workflows, we would like to learn about a complex simulator that describes a physical system, in nearly every case imperfectly [4,5]. These simulations are usually computationally intensive, high dimensional and the outputs are very sensitive to the inputs, making it hard to use them directly to compare with observations.

To overcome these challenges, we use a surrogate model approach based on a GP emulator [6]. We run a limited sample of points based on an experimental design over the input space, and fit the GP to the simulation outputs. The GP is then queried for a large number of input points from the experimental design and an approach known as history matching is used to compare with the observations to calibrate the model. The result from this is a set of input points that are plausible given the observations and all uncertainties. In the following, we describe the steps in this workflow in more detail.

### (a) Experimental design

Based on the input parameters, we first need to specify a way to choose points at which to run the simulator. This is done via an experimental design, which is specified based on a probability distribution from which each individual input parameter is drawn independently. Based on these distributions, the simplest approach is to use Monte Carlo sampling to pick random input points to run. However, for expensive computational models the number of inputs is often limited, so in practice a more common approach is to choose the design in a way that attempts to maximize the accuracy of the underlying approximation. This can be further split into two approaches: one-shot designs, that choose all simulation points at once [14], or sequential designs that iteratively choose the next best point to simulate based on the existing information [15].

In this study, we use a one-shot design based on a Latin hypercube sampling approach [14]. In a Latin hypercube, we guarantee that we draw from all quantiles of each underlying parameter. In other words, for a design with 4 points, a Latin hypercube will ensure that the four chosen points are each from a different quartile of each underlying parameter. The exact choice of values is done randomly within this constraint, so Latin hypercube designs do have some variability associated with them.

For small designs, Latin Hypercubes have been shown to perform better than Monte Carlo sampling under certain circumstances [14], though they are usually not as effective as sequential designs. However, because of their simplicity, we use them in this example as a straightforward way to draw samples for building a surrogate model of the underlying simulator.

### (b) Gaussian process emulator

To fit our surrogate model, we use a GP emulator to approximate the simulation. A GP is a non-parametric model for regression that approximates the complex simulator function as a multivariate normal distribution. Because the simulator is deterministic, a GP interpolates between the known simulation points in a robust way and provides uncertainty estimates for any predictions that it makes. Because it has an uncertainty estimate, it is commonly used in UQ workflows [4,15,16].

A GP is specified by a mean function and a covariance function. We use a zero mean GP with a Squared Exponential Kernel in this example, though more complicated mean functions and covariance kernels are common, particularly if we have some underlying knowledge of the shape of the simulator output. The squared exponential kernel is defined as

2.1 $$K(x,x^{\prime })=\sigma^{2}\exp\left(-\sum_{i}\frac{{\left(x_{i}-x_{i}^{\prime }\right)}^{2}}{2\theta_{i}^{2}}\right),$$

where *x*_*i*_ is the *i*th input parameter, *σ* is an overall covariance scale, and *θ*_*i*_ is a correlation length associated with the *i*th input. These hyperparameters *θ*_*i*_, *σ* are estimated based on the data.

To predict the function and its uncertainty at unknown points, the covariance matrix must be inverted. The posterior mean and variance (i.e. once hyperparameter values are chosen) at the unknown point $x^{*}$ given a set of *n* inputs *x* and simulator outputs *y* are computed via

2.2 $$\begin{array}{rl} & m(x^{*})=K(x^{*},x)K{\left(x,x\right)}^{-1}y\\ \text{and}\;\; & V(x^{*})=K(x^{*},x^{*})-K(x^{*},x)K{\left(x,x\right)}^{-1}K(x,x^{*}), \end{array}\}$$

where *K*(*x*, *x*) is the *n* × *n* matrix of the covariance kernel evaluated at all pairs of points. Because the kernel is positive definite, this inversion is done by Cholesky decomposition, requiring $O(n^{3})$ operations. Once the covariance matrix is inverted and cached, mean predictions require O(n) operations while variance predictions require $O(n^{2})$ operations.

In order to make predictions, we need to fit the hyperparameter values for *θ*_*i*_ and *σ*. A common approach is to use the maximum marginal likelihood, which is easy to compute for a GP once the covariance matrix has been factorized

2.3 $$\log p(y|x)=-\frac{1}{2}y^{T}K{\left(x,x\right)}^{-1}y-\sum_{i}\log L_{ii}-\frac{n}{2}\log2\pi ,$$

where *L* is the factorized covariance matrix using Cholesky decomposition. This finds a set of correlations lengths and the overall covariance scale, and these parameters can be used to predict the value of the function at unknown points.

While the simulator is deterministic and we thus should theoretically be able to use equation (2.1) directly, in practice numerical round-off errors can cause the Cholesky factorization to be unstable. To mitigate this, a ‘nugget’ term is added to the diagonal that adds a small amount of noise to stabilize the matrix inversion [17]. There are several ways to estimate the nugget: it can be fixed (known noise level), it can be fit as an additional hyperparameter, or it can be found adaptively by factorizing the matrix with increasing noise levels until the algorithm succeeds. In this example, we use the adaptive approach as we find it tends to be a very robust way to fit an emulator with a small nugget.

In this example, we focus on a computer simulation with a single output for the sake of simplifying the presentation. However, real UQ problems typically involve multiple observations and simulation codes that produce multiple outputs as well. These could include multiple observable fields, as well as spatially- or time-varying fields. In this case, the overall UQ procedure is the same, but multiple surrogate models must be fit that predict all of the output quantities of interest. The simplest way to handle multiple outputs is to fit an independent emulator to each quantity of interest (which can be done in parallel using mogp_emulator). However, this approach will fail to capture the correlation structure present in the outputs. One way to mitigate this problem is to perform a dimension reduction on the outputs and emulate the reduced set of outputs [18] which simultaneously reduces the computational cost of fitting and ensures that samples drawn from the emulators more closely resemble the simulation output. However, the overall procedure for fitting an individual emulator remains identical regardless of the total number of outputs.

### (c) History matching

Once we have predictions for a large number of query points, it is straightforward to compare with observations. History matching is one way to perform this comparison [16]—in history matching, we compute an implausibility metric *I* for each query point by determining the number of standard deviations between the observation and the predicted mean from the approximate model

2.4 $$I(x^{*})=\frac{|z-m(x^{*}))|}{\sqrt{\sigma_{z}^{2}+V(x^{*})+\sigma_{d}^{2}}},$$

where *z* is the observed quantity and *σ*_*z*_ is its observational error (as a standard deviation) and *σ*_*d*_ is the model discrepancy, described below. We can then ‘rule out’ points that are many standard deviations from the mean as being implausible given the observation and all sources of error.

As noted above, there are three types of uncertainty that we need to account for when computing implausibility:

(i) Observational error, which is uncertainty in the observed value itself;
(ii) Uncertainty in the approximate model, which reflects the fact that we cannot query the full computational model at all points; and
(iii) Model discrepancy, which is uncertainty about the model itself, and measures how well the computational model represents reality.

In practice, (i) and (ii) are straightforward to determine, while (iii) is much trickier [19]. However, studies have shown that not accounting for model discrepancy leads to overconfident predictions, so this is essential to consider to give a thorough UQ treatment to a computational model. However, estimating model uncertainty is in itself a difficult (and often subjective) task, and is beyond the scope of this tutorial, as it requires knowledge about the approximations made in the simulation. Thus, we will restrict ourselves to only accounting for uncertainty in the approximate model in this tutorial, but note that realistic UQ assessments require careful scrutiny and awareness of the limitations of computational models.

An alternative approach to history matching for model calibration is to perform a full Bayesian model calibration [4], which aims to compute the posterior distribution of the simulator inputs conditioned on the observational data and the points at which the simulator was evaluated. As with history matching, this approach uses a fast surrogate model to approximate the simulator and accounts for all errors including observational error and model discrepancy. It has the advantage of generating a probability distribution (or samples drawn from one in most practical cases) for the parameter values, while history matching is only able to determine if points can be ruled out or not. However, full Bayesian calibration requires that the emulator has a low uncertainty over the entire input space to prevent emulator uncertainty from dominating the calibration results. This condition is frequently not met given the high-dimensional parameter space and computational costs of running many simulations in most practical applications. History matching can still produce useful output with an imperfect emulator, as it simply will be unable to rule out points in regions of space where the emulator uncertainty is too large, while still giving useful information in other parts of parameter space. Thus, because of its robustness to emulator uncertainties, we focus on history matching in this example.

In situations where the simulation has multiple outputs, history matching requires a method for combining the implausibility measure for multiple outputs into a single implausibility metric for the given simulator input. This is usually done by taking the second or third highest individual implausibility metric value to avoid a situation where poor performance of the emulator for a particular output causes a point that is otherwise a good fit to the data from being ruled out [20]. Otherwise, the history matching procedure is the same regardless of the number of simulation outputs.

### (d) Implementation with mogp_emulator

The above components are implemented in the mogp_emulator software library, which is written in Python and builds on the Numpy and Scipy libraries [21,22] to handle the array operations and linear algebra, and probability distributions and optimization libraries, respectively. The library is released under an MIT license and is under continued development. The package includes a number of features not used in this example, including flexible mean function specification, prior distributions for maximum a posteriori estimation for the GP emulators, and additional experimental design procedures.

## 3. Earthquake model

As a concrete example of a complex physical simulator, we examine an earthquake rupture simulation [2,11]. In seismology, the most basic quantity that we can measure about an earthquake is its size, quantified by the seismic moment. The seismic moment is proportional to the relative displacement across the two sides of the fault (known as the slip) multiplied by the area of the fault plane that experienced this slip and a modulus of rigidity. Larger earthquakes occur when either more slip occurs or the area that slipped increases (in nature, these two quantities are correlated so earthquakes get bigger by both increasing the slip and the area simultaneously).

### (a) Dynamic earthquake rupture

Earthquake slip can be computed by solving the elastic wave equation coupled to a frictional failure model on the fault [2]. The simulation calculates the size of an earthquake (which can be measured from seismic data) [23] given an initial stress tensor in the material (a quantity that is poorly constrained from seismic data). The simulation computes the earthquake size based on the stress tensor combined with the fault geometry and frictional failure properties, both of which are taken to be known here for the sake of simplicity.

**Figure 1.**
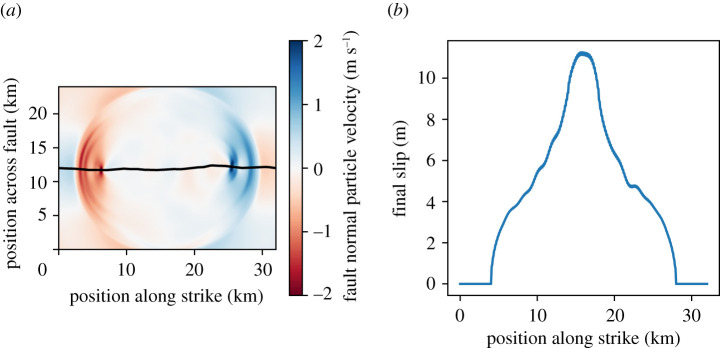
(*a*) Snapshot of an earthquake simulation. The bumpy dark line is the fault surface. The colour scale represents the ground motions from the resulting earthquake as the elastic waves carry the stress changes from the slip propagation through the medium. (*b*) Final slip at the end of a simulation. We compute the seismic moment by integrating the final slip as a function of space. (Online version in colour.)

Physically, slip occurs when the shear stress on the fault exceeds the fault strength. Fault strength is determined by a friction law that compares the shear force on a patch of the fault to the normal force acting on that patch of the fault [24]. When this condition is met, the fault slips on this local patch, which changes the forces acting on the other fault patches based on the elastic wave equation. Thus, to make a physical model of an earthquake, we need to specify the initial forces on the fault, the strength of the fault and the elastic medium surrounding the fault. In general, the initial forces on the fault cannot be determined in the earth [25], and we will use a UQ workflow to try and estimate these quantities. A snapshot of the ground shaking from one of the simulations is shown in figure 1—the bumpy line is the rough fault surface, and the colour scale shows the propagation of elastic waves away from the fault due to the slip on the fault.

Complicating matters is the fact that earthquake faults are not smooth planes, but instead rough bumpy surfaces with a fractal geometry [26]. An important consequence of this is that the smallest wavelength bumps have the largest effect on the resulting forces [27]. This is what makes earthquake problems challenging to model: at a given model resolution, the simulation is omitting details that play an important role. This small scale roughness that is left out of the model must instead be accounted for when setting the strength of the fault. However, for this demonstration, we will assume that both the rough geometry of the fault and the fault strength are known in advance, and it is just the initial stress (forces) that must be inferred.

### (b) Simulation details

The simulation requires that we specify the initial stress tensor acting on the earthquake fault in order to run a simulation. For this case, we run a two-dimensional plane strain simulation of a fault that is 32 km in length to reduce the problem to a reasonable computational level such that it only takes a short amount of time to run. In a plane strain model, the elastic wave equation can be written in velocity/stress form as

3.1 $$\begin{array}{rl} \rho \frac{\partial v_{x}}{\partial t} & =\frac{\partial \sigma_{xx}}{\partial x}+\frac{\partial \sigma_{xy}}{\partial y}\\ \rho \frac{\partial v_{y}}{\partial t} & =\frac{\partial \sigma_{xy}}{\partial x}+\frac{\partial \sigma_{yy}}{\partial y}\\ \frac{\partial \sigma_{xx}}{\partial t} & =\lambda \left(\frac{\partial v_{x}}{\partial x}+\frac{\partial v_{y}}{\partial y}\right)+2G\frac{\partial v_{x}}{\partial x}\\ \frac{\partial \sigma_{yy}}{\partial t} & =\lambda \left(\frac{\partial v_{x}}{\partial x}+\frac{\partial v_{y}}{\partial y}\right)+2G\frac{\partial v_{y}}{\partial y}\\ \text{and}\;\;\frac{\partial \sigma_{xy}}{\partial t} & =G\left(\frac{\partial v_{x}}{\partial y}+\frac{\partial v_{y}}{\partial x}\right) \end{array}\}$$

where *v*_*x*_ and *v*_*y*_ are the particle velocity components, *σ*_*xx*_, *σ*_*yy*_ and *σ*_*xy*_ are the three stress tensor components (two compressive and one shear), *ρ* is material density, *λ* is the first Lamé parameter and *G* is the shear modulus.

Frictional failure follows the slip weakening friction law [24], where the friction coefficient *μ* depends on the fault slip *U* as

3.2 $$\mu (U)=\left\{ \begin{array}{ll} (1-U/D_{c})(\mu_{s}-\mu_{d})+\mu_{d} & \left(U<D_{c}\right)\\ \mu_{d} & (U\geq D_{c}). \end{array}\right.$$

Here, *μ*_*s*_ is the static friction coefficient, *μ*_*d*_ is the dynamic friction coefficient and *D*_*c*_ is the slip scale over which friction transitions from static to dynamic. The simulation is initiated at a fixed point at the centre of the fault by increasing the shear stress to the failure level over a patch of width 4 km. Strong barriers arrest rupture 2 km from the ends of the simulation, which caps the maximum size of the earthquake. All simulation parameters are specified in table 1.

**Table 1.** Base earthquake model parameter values.

parameter	value
*ρ*	2.68 × 10^3^ kg m^−3^
*λ*	32.04 GPa
*G*	32.04 GPa
*μ*_*s*_	0.7
*μ*_*d*_	0.2
*D*_*c*_	0.8 m

The fault profile is generated following a fractal geometry by creating a self-similar power spectrum in Fourier space with random phase and taking the real part of the fast Fourier transform and removing the linear trend. The RMS deviation from planarity is fixed to be smaller than the fault length by a factor of 10^−2^, which is typical for natural faults [28]. Roughness is cut off at wavelengths shorter than 20 times the grid spacing. We have run the analysis on several realizations of the rough fault profile and find that the general conclusions are not sensitive to the exact choice of fault geometry. Changing the profile does influence the exact values of the simulator output, but the results of the UQ analysis are largely the same in that the history matching procedure is able to rule out much of the parameter space.

*σ*_*yy*_ describes the normal force on the fault, and *σ*_*xx*_ describes the normal force in the orthogonal direction. The shear component *σ*_*xy*_ sets the shear force acting on the fault. Note, however, that all three components matter because the fault is not a perfect plane, and we must project the tensor into the local shear and normal components for a given patch on the fault to determine the actual forces on the fault. While we do not know the exact values of the stresses on earthquake faults, we do know a few general things that we should incorporate into our simulations:

(i) Pressure increases linearly with depth due to the weight of the rocks. This can be mediated by fluid pressure counterbalancing some of the overburden pressure, and earthquakes start at different depths, so we are not sure of the exact value. However, at typical depths where earthquakes start (5–10 km), this pressure is expected to be somewhere in the range of −80 MPa to −120 MPa (stress is assumed to be negative in compression). Therefore, we can use this range to choose values for one component, and then assume that the other component is similar (say ±10% of that value).
(ii) Shear stresses are below the failure level on the fault. This can be understood as simply reflecting that earthquakes tend to start in one place and then grow from there, and do not start in many places at once. Thus, we will assume that since the frictional strength of the fault in our simulation is 0.7 times the normal stress, the initial shear stress is between 0.1 and 0.4 of the normal stress.

Thus, we parametrize the simulations with three inputs: a normal stress that is uniformly distributed from −120 MPa to −80 MPa, a shear to normal ratio uniformly distributed from 0.1 to 0.4, and a ratio between the two normal stress components uniformly distributed from 0.9 to 1.1. These three parameters can be sampled via any experimental design approach described in §2a.

To run the earthquake simulations, we use the fdfault application. fdfault is a high performance, parallelized finite difference code for simulation of frictional failure and wave propagation in elastic-plastic media. It features high order finite difference methods and is able to handle complex geometries through coordinate transformations and implements a provably stable method [29].

Our simulations use a 401 by 302 point computational grid, with co-located points along the rough fault interface representing the displacement discontinuity across the fault surface. The time step is chosen based on a Courant–Friedrichs–Lewy ratio of 0.3 based on the minimum grid spacing and the shear wave speed in the material. Our simulations use 800 time steps to ensure that all ruptures have sufficient time to rupture until they arrest, either due to encountering an unfavourable fault orientation or reaching the edge of the fault. On a 4 core MacBook, these simulations take about 20 s each using four processors. These parameters were chosen within the constraints of the tutorial time slot to make the problem practical.

## 4. Simulation management

**Figure 2.**
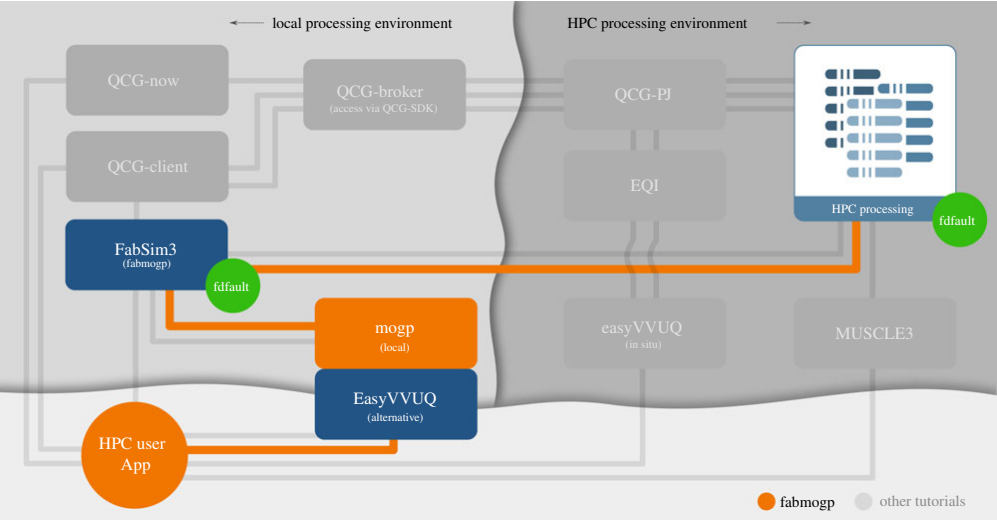
Illustration of the workflow used in our simulations. Local resources are shown on the left in light grey, and remote HPC resources on the right in darker grey. The HPC user (orange circle in the lower left corner) uses a local machine running mogp_emulator to set up the UQ workflow. This connects with FabSim3 on the local machine, which is running the fabmogp plugin. The plugin connects via SSH to the cluster, where it runs the fdfault simulations (though in practice, these are actually run locally in our tutorial). FabSim3 collects the results back on the local machine, where mogp_emulator performs the surrogate modelling and history matching. Other workflows enabled by the VECMA toolkit are shown in grey boxes on the local and HPC machines. (Online version in colour.)

The UQ workflow described above can be run via mogp_emulator, while the parallel earthquake simulations would need to be run manually. However, in practice, this is challenging and makes simulations difficult to reproduce. Thus, in our implementation, we have written a plugin for FabSim3 which we call fabmogp to automate the various steps in the workflow. A map illustrating where the different software components reside on local and remote resources is shown in figure 2, which also shows where additional components not used here would reside. In this illustration, the local resources are shown to the left, while the remote HPC resources are shown to the right, and the connections used in our workflow are shown in orange. Our workflow involves the user (lower left corner) running mogp_emulator on the local machine and using FabSim3 (via the fabmogp plugin) to run the ensemble on the remote resource. However, in practice, our simulations are small enough that this can also be run on the local machine. FabSim3 then collects the results back onto the local machine, where the UQ analysis is performed. Other workflows supported by the VECMA toolkit are shown in the other grey boxes throughout the diagram.

FabSim3 is a toolkit for user-developers to help automate computational workflows involving many simulations and remote resources. It has been used in a variety of disciplines, for instance to facilitate coupled atomistic/coarse-grained materials simulations and to perform large-scale sensitivity analysis of agent-based migration models [10]. The tool is open-source (BSD 3-clause license) and one of the main components of the VECMA toolkit.

We conduct our simulations using two FabSim3 simulation tasks: mogp_ensemble and mogp_analysis. The mogp_ensemble workflow will automatically sample the Latin hypercube to create the desired number of points, set up all of the necessary earthquake simulations, and run them. The advantage of using this approach over the manual approach described above is that the runs are each performed in individual directories, with input, output and environment curated accordingly. This makes it very easy to reproduce individual runs, and also helps with the diagnostics in case some of the simulations exhibit unexpected behaviours.

Additionally, our choice of earthquake simulation has made a number of compromises in order to ensure that the simulations run in a reasonable amount of time given the constraints of the workshop format where it was initially presented. However, to make the simulations more realistic will require additional computational resources. A typical three-dimensional dynamic rupture simulation of a similarly sized earthquake will usually require tens of hours on 64+ cores, depending on the exact model set-up and simulation approach used. By implementing this workflow using FabSim3, we are able to test and debug the simulations locally, yet we can easily scale the simulations up to larger problems in three dimensions on a cluster without needing to change any of our execution scripts. This illustrates the utility of using a simulation management tool like FabSim3.

Once the ensemble has run, FabSim3 can automatically fetch the simulation results for analysis. The analysis to fit the GP emulator and perform history matching is implemented in a FabSim3 task to collect the simulation results and perform the UQ workflow.

## 5. Results

### (a) Simulator runs

A sample output from the Latin hypercube design with 20 sample points is shown in table 2. The input parameters take on a range of values spread out through the entire space, which are converted into the raw stress values for execution in the fdfault simulation.

**Table 2.** Latin hypercube experimental design samples used for building the surrogate model and the corresponding integrated slip

normal stress	shear/normal stress	normal stress ratio	simulator output
−89.31 MPa	0.365	1.090	213.37 m km
−112.29 MPa	0.213	0.976	110.72 m km
−117.27 MPa	0.177	1.019	69.60 m km
−90.26 MPa	0.194	0.915	54.71 m km
−97.72 MPa	0.261	1.007	145.46 m km
−115.89 MPa	0.373	1.077	289.05 m km
−103.42 MPa	0.137	0.988	45.72 m km
−101.81 MPa	0.122	1.052	41.91 m km
−92.34 MPa	0.310	0.926	175.67 m km
−109.73 MPa	0.318	0.908	219.51 m km
−106.26 MPa	0.395	1.048	296.45 m km
−119.08 MPa	0.173	0.940	68.19 m km
−83.20 MPa	0.102	0.968	29.24 m km
−84.33 MPa	0.291	1.020	145.85 m km
−104.50 MPa	0.152	0.992	51.69 m km
−111.31 MPa	0.222	1.099	127.13 m km
−86.18 MPa	0.349	0.953	191.35 m km
−81.37 MPa	0.270	1.039	123.67 m km
−94.62 MPa	0.247	0.944	123.76 m km
−99.95 MPa	0.330	1.062	211.74 m km

The simulator output is calculated by integrating the final slip at the end of the simulation over the entire fault plane using Simpson’s rule. Because all simulations have the same shear modulus and our simulations are two dimensional, we simply use this integrated slip as the simulator output as it is proportional to the seismic moment. Values range from 35 m km to around 300 m km.

### (b) Surrogate model

From these simulator runs, we fit a GP emulator to the outputs using the default mogp_emulator parameters. We use the SciPy implementation of L-BFGS-B [22,30] to minimize the negative marginal log-likelihood, and use gradient information as the log-likelihood gradient can be computed in closed form and requires little computational overhead beyond performing the Cholesky decomposition that is cached from the log-likelihood computation [6].

Because the hyperparameters are constrained to be positive, we fit the logarithm of the correlation lengths and overall covariance to convert the problem into an unconstrained optimization, which tends to be more stable. The resulting correlation lengths on a linear scale are 43.755, 0.109 and 0.599, for the normal stress, shear to normal, and normal stress ratios, respectively. The overall covariance is 131.994, which has also been converted to a linear scale. The covariance scale matches the range of simulations output noted in table 2, and the correlation lengths are of a similar scale to the actual input values, suggesting that our emulator does a reasonable job of capturing the information in the simulation outputs.

### (c) Validation

We validate the surrogate model by drawing a separate Latin hypercube sample with 10 design points (table 3). We note that while in many other statistical techniques it is common to withhold a subset of the training data for validation purposes, the space-filling nature of the Latin hypercube suggests it is best to draw two separate samples to ensure that both the training and validation data aim to cover the input space as uniformly as possible. Once we have run the additional simulations, we validate the emulator by computing the predicted means and variances and comparing with the actual simulated values by computing the standard error (difference between the predicted mean and the actual value normalized by the prediction standard deviation). For a valid emulator, we expect most of the standard error values to lie within ±3 standard deviations from the mean. Other metrics can be used to validate emulators [31] that produce a single validation metric for the entire validation set rather than an individual metric for each validation point.

**Table 3.** Latin hypercube experimental design samples used for validating the surrogate model and the corresponding integrated slip

normal stress	shear/normal stress	normal stress ratio	simulator output
−116.47 MPa	0.160	1.001	62.21 m km
−82.40 MPa	0.200	1.035	51.63 m km
−101.98 MPa	0.311	0.931	197.19 m km
−88.25 MPa	0.104	0.942	31.59 m km
−94.82 MPa	0.274	0.910	149.74 m km
−114.32 MPa	0.299	0.968	212.81 m km
−97.04 MPa	0.384	1.058	247.63 m km
−106.13 MPa	0.227	1.000	122.31 m km
−108.90 MPa	0.353	1.092	253.26 m km
−86.82 MPa	0.149	1.063	39.38 m km

**Figure 3.**
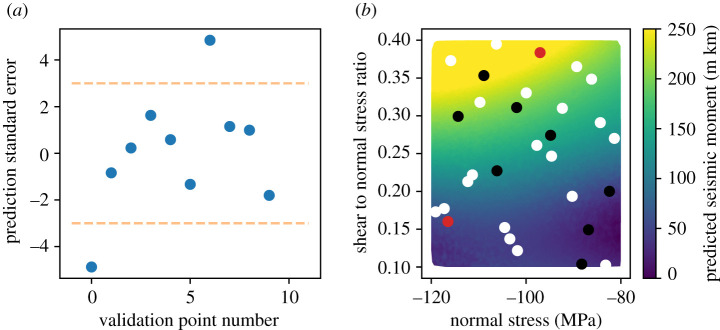
(*a*) Emulator validation results for the 10 validation points drawn using a Latin hypercube experimental design. We find that the emulator provides valid predictions for 8/10 validation points. (*b*) Spatial distribution of the training and validation points, projected into the normal stress and shear to normal stress ratio plane (the simulator output is not highly sensitive to the additional normal stress ratio). The background colour scale shows the predicted emulator mean and illustrates the approximate behaviour of the underlying simulator. The output is most strongly dependent on the shear to normal stress ratio. White points are the training samples, black points are validation points where the emulator performance is valid, and red points are the two points where a validation failure occurs. We note that the failure points are near the transitions between regions where the dependence of the output to the underlying input is strongly varying. This suggests that the underlying function is non-stationary, and the resulting emulator is overconfident in the predictions. However, we find that despite these validation failures the emulator is still providing enough useful information to proceed with the analysis due to its accuracy over the majority of the input space. (Online version in colour.)

The values of the standard error for the 10 validation points are illustrated in figure 3*a*. We find that 8 of the 10 validation points lie within the 3 s.d. window, while the remaining 2 points are outside of this range and indicate that the emulator is not perfectly reproducing the underlying function. There are several potential causes for these types of failures, which can be illustrated by looking at the spatial distribution of the training and validation points in figure 3*b*. Figure 3*b* shows the input space projected into the normal stress-shear to normal stress plane, which are the two inputs to which the simulator output is most sensitive. The background colour scale shows the emulator predictions for a much larger set of 10 000 sample points. The simulation is most sensitive to the shear to normal stress ratio, with low values indicating rupture arrest and high values indicating rupture propagation. The white points are the 20 training points, the black points are the eight validation points where the emulator predictions are valid, and the two red points are the validation failures.

For this problem, the emulator validation failures occur because in some portions of parameter space the simulator output is very sensitive to the inputs, while simultaneously there are other parts of the parameter space where the output is not very sensitive to the inputs. Our emulator uses a squared exponential covariance kernel, which is an example of a stationary covariance kernel in that it assumes a uniform correlation length should apply throughout the entire parameter space. For many nonlinear simulators, this assumption does not hold, and the resulting emulator will have some regions of the input space where it does not provide good predictions. In this case, the emulator is overconfident in the regions where the validation failures have occurred, but results in good performance for most of the input space.

To correct this validation failure, we would first consider building an emulator with a more informative mean function, as in many cases a mean function that better captures prior information about the shape of the output will alleviate problems of a non-stationary underlying simulator. Prior distributions on the hyperparameters can also provide additional constraints if the experimental design does not sample the input space well enough to robustly estimate the hyperparameter values. Drawing additional input samples can also help if there are a few problem areas where the emulator performs poorly and can be improved by constraining the value of the underlying simulator. We have experimented with training sets with 50 points and find that the emulator performs better in the sense that we observe a decrease in the prediction uncertainties. However, validating the emulator shows there are still regions where the predictions are overconfident due to non-stationarity of the underlying function, suggesting that an approach using a mean function is required to overcome this problem. However, we feel that a careful exploration of this issue is beyond the scope of this work, and thus we proceed with the original emulator as fit in the tutorial to perform history matching.

### (d) History matching

With the fit GP emulator, we can now make predictions using a dense sampling of points drawn from the experimental design and compare with an observed value using history matching. We use 10 000 samples in the analysis that follows. For the sake of this demonstration, we simply choose an arbitrary value from within the range of simulation outputs to serve as our ‘observed’ value to illustrate how the procedure works, though in practice the observed seismic moment would be the size of a particular earthquake on the fault that is being studied. We assume that there is no error in the true value and the only uncertainty is the emulator prediction uncertainty to simplify the demonstration, though in practice the additional uncertainty from the observational error and model discrepancy will simply expand the size of the space that has not been ruled out.

**Figure 4.**
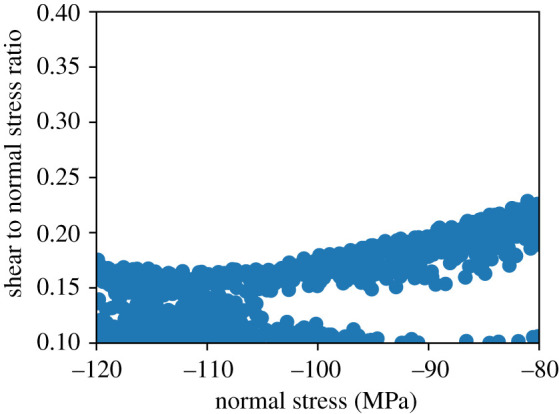
Points that have not been ruled out yet (NROY) projected into the normal and shear/normal plane of the parameter space. Note that the points are fairly tightly clustered along a line, showing that the earthquake size is very sensitive to the stress tensor components. (Online version in colour.)

An example of the samples that have been not ruled out yet (NROY) for the observed value of 58 m km is shown in figure 4 projected into the normal and shear/normal ratio plane of parameter space. We use a plausibility threshold of 3 s.d. from the mean to rule out points. We note that the NROY space is fairly clustered along a specific curve in this space. At high compressive normal stresses, this seismic moment is produced for shear/normal stress ratios of around 0.16, while at lower normal stresses the shear/normal stress must be slightly higher near 0.2 to produce the known value. At very low shear to normal stresses, there is a region that cannot be ruled out, though the fact that this occurs near the boundary of the space suggests this may be an artefact of our original sampling. Designs with 50 sample points do not exhibit this feature. We note that the projection shown in figure 4 was found to capture most of the structure in the space, and suggests that the additional normal stress component is less important for predicting the final seismic moment in our simulations.

**Figure 5.**
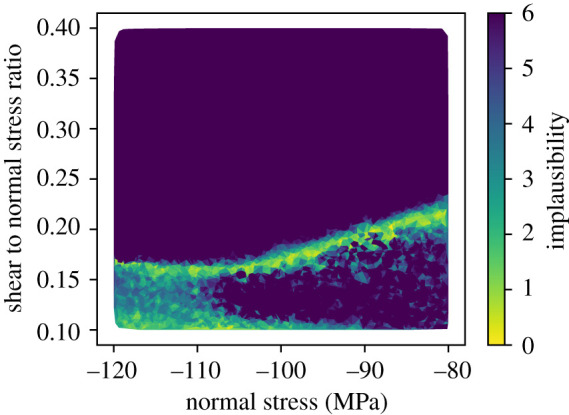
Implausibility metric (number of standard deviations between the observation and the predictions of the surrogate model, equation (2.4)) in the parameter space projected into the normal and shear/normal plane. As with the NROY plot, this illustrates the high sensitivity of the output to the stress components. (Online version in colour.)

The implausibility metric used to determine the NROY space (equation (2.4)) is shown in figure 5. As we can see, most values that are ruled out have implausibility metrics much greater than 6, indicating that we have a high degree of confidence that they can be ruled out. This knowledge allows us to focus further simulation effort and analysis on a much narrower part of parameter space, so that future computational effort is focussed on the most likely parameter values to improve our understanding of the problem and make predictions.

## 6. Conclusion

This paper and the associated tutorial demonstrate the use of a variety of computational tools to implement and execute a UQ calibration workflow on a computationally intensive earthquake model. Our implementation can automate the entire workflow with a few simple command line instructions, and the FabSim3 plugin facilitates scaling our simulations to more intense problems requiring execution on a computer cluster. Our implementation also makes the UQ and earthquake simulation methods accessible to new users and facilitates reproducibility by providing the computational environment required to run them. Our results also illustrate how the mogp_emulator package allows implementation of robust model calibration approaches on problems that have not previously considered such an approach. The library allows flexible specification of all components of the calibration workflow and is easily adaptable to other physical systems.

There are numerous challenges in applying these methods to more complex research problems, some of which we have highlighted in this paper. In particular, the computational effort required to carry out the training and validation simulations will typically be much larger and require a high-performance computing cluster. One advantage of our approach is that if the workflows have been appropriately defined using FabSim3 for simulation management, the same scripts can be used to run the ensemble on the cluster that were used here, only requiring a larger computational expense. The exact number of simulator samples that should be run will depend on the computational expense and resources available, but will also depend on the number of input parameters in the problem due to the well-known ‘curse of dimensionality’ in that the size of the input space grows exponentially with the number of parameters. Thus, more realistic problems may require a larger number of simulator runs to obtain emulators with sufficiently good performance.

Once the sample points are drawn from the simulator, more realistic problems will require more computational effort to fit the surrogate emulators to the data. This can be due to multiple outputs and observations, which require multiple emulators be fit to the simulator outputs and thus increase the computational cost. However, dimension reduction techniques [18] for handling multiple outputs can reduce the cost of fitting the emulator by only fitting a few principal components to ensure that the emulators capture the correlation structure in the simulators. Additionally, more input parameters can increase the fitting costs due to the additional correlation lengths that must be estimated, as minimization algorithms tend to be slower to converge for high-dimensional search spaces. Once the emulators are fit, prediction costs will also scale with the number of emulators, and high-dimensional input spaces will also require more query points to sample the input space at a high enough density to be able to rule out parts of the space. These factors will tend to increase the cost of fitting the surrogate models relative to this example, though in general for most applications the cost of running the simulator remains the largest computational expense.

The UQ results demonstrate that given the seismic moment of an event, we can rule out much of the input stress parameter space, as the earthquake size is highly sensitive to the stress. This can potentially overcome one of the main challenges of using dynamic earthquake modelling for seismic hazard analysis. In probabilistic seismic hazard analysis [32], the standard approach for estimating risk due to strong ground motions, analysts must first determine the distribution of earthquake sizes expected to occur over a given time period. This is usually done empirically based on very limited observations, and does not attempt to determine if such earthquake sizes are consistent with physical models. Our UQ approach could enable use of dynamic simulations in this approach by providing a set of NROY points that are consistent with the limited observations, and use those points to simulate a much more comprehensive set of ruptures consistent with the historical data to supplement the limited existing strong motion records [33]. These physical simulations can thus capture the natural variability of events in a region, something that current empirical approaches cannot do in a physical way. This will enable physics-based seismic hazard analysis that exploits simulations in a way not previously possible, and give more robust estimates of future earthquake sizes and ground motions in order to better constrain uncertainties in both the physical models and the predicted hazard.

## Data accessibility

All simulation codes used in this work are publicly available on Github under free and open source software licenses:

— mogp_emulator https://github.com/alan-turing-institute/mogp-emulator
— FabSim3 https://github.com/djgroen/fabsim3
— fdfault https://github.com/edaub/fdfault

The FabSim3 plugin that implements all of the simulations and analysis described in this work is also available under a free and open source license on Github:

— fabmogp https://github.com/alan-turing-institute/fabmogp

The original tutorial that describes this workflow, the underlying mogp_emulator code and the FabSim3 commands can be found at https://github.com/alan-turing-institute/vecma_workshop_tutorial. The computational environment and associated scripts and build instructions used to produce the simulations, figures and the typeset manuscript is available as a Docker image in a Github repository. The repository contains detailed instructions on building and running the simulations and is available at https://github.com/alan-turing-institute/fabmogp_paper. The reproducibility of our work can be confirmed by running the simulations as described in this repository and then computing cryptographic hash functions using the repro-catalogue tool (https://github.com/alan-turing-institute/repro-catalogue). We include our hash values in the repository, and the repro-catalogue tool can compare the obtained hash values with our results.
